# Potential types of bias when estimating causal effects in environmental research and how to interpret them

**DOI:** 10.1186/s13750-024-00324-7

**Published:** 2024-02-07

**Authors:** Ko Konno, James Gibbons, Ruth Lewis, Andrew S Pullin

**Affiliations:** 1 School of Natural Sciences, Bangor University, Bangor, UK; 2 School of Medical and Health Sciences, Bangor University, Bangor, UK

**Keywords:** Critical appraisal, Risk-of-bias assessment, Validity of causal inferences, Threats to internal validity

## Abstract

To inform environmental policy and practice, researchers estimate effects of interventions/exposures by conducting primary research (e.g., impact evaluations) or secondary research (e.g., evidence reviews). If these estimates are derived from poorly conducted/reported research, then they could misinform policy and practice by providing biased estimates. Many types of bias have been described, especially in health and medical sciences. We aimed to map all types of bias from the literature that are relevant to estimating causal effects in the environmental sector. All the types of bias were initially identified by using the Catalogue of Bias (catalogofbias.org) and reviewing key publications (*n* = 11) that previously collated and described biases. We identified 121 (out of 206) types of bias that were relevant to estimating causal effects in the environmental sector. We provide a general interpretation of every relevant type of bias covered by seven risk-of-bias domains for primary research: risk of confounding biases; risk of post-intervention/exposure selection biases; risk of misclassified/mismeasured comparison biases; risk of performance biases; risk of detection biases; risk of outcome reporting biases; risk of outcome assessment biases, and four domains for secondary research: risk of searching biases; risk of screening biases; risk of study appraisal and data coding/extraction biases; risk of data synthesis biases. Our collation should help scientists and decision makers in the environmental sector be better aware of the nature of bias in estimation of causal effects. Future research is needed to formalise the definitions of the collated types of bias such as through decomposition using mathematical formulae.

**Supplementary Information:**

The online version contains supplementary material available at 10.1186/s13750-024-00324-7.

## Background

Estimation of average causal effects of interventions or exposures can provide important evidence to inform environmental policy and practice. There is a risk that if these estimates are derived from poorly conducted (or selective reporting or selectively reported [1–4]) primary (e.g., impact evaluation studies) or secondary research (e.g., evidence reviews), then they will potentially misinform policy and practice. Biased estimates of average causal effects (inaccuracy) are more problematic than imprecise estimates of average causal effects due to random error which may be measured by standard errors or confidence intervals [5] because a bias is a systematic error or ‘deviation from the truth’ [6, 7], and can be a direct threat to the validity of a causal inference [8]. Biases can be quantified only when the true average causal effects are known, or when hypothetical true (or expected) average effects are set as reference values (i.e., *B* = *m* − *µ*, where *m* is an estimated average causal effect and *µ* is the true causal effect [7, 9, 10]; it is also defined as $E\left( {\widehat{\theta }} \right) - \theta$ [8]). This means that, when there is a systematic error, an estimated average causal effect is inaccurate and either an under- or overestimate of the true average causal effect (i.e., *m* = *µ* ± *B*, where B ≠ 0 [10]). However, distinguishing biases from random errors and quantification of biases may not be possible using real-world data [6]. Instead of quantifying biases, the extent of ‘risk of bias’ or ‘threats to internal validity’ may need to be communicated explicitly [11–13].

Many types of bias (specific phenomena resulting in systematic errors described in the literature and on the Internet) have been described, most notably in medical and health sciences where methods for assessing the risk of bias originated (e.g., Catalogue of Bias describes 62 types of bias as of 29 August 2021: catalogofbias.org). For example, observer bias is described as ‘*the process of observing and recording information which includes systematic discrepancies from the truth*’ [14], and detection bias is described as ‘*systematic differences between groups in how outcomes are determined*’ [15]. Types of bias, which are considered by individuals or groups as distinctive phenomena that can give rise to biased estimates, are generally described freely, but some can also be defined formally using mathematical languages [e.g., confounding bias and collider bias are often defined using directed acyclic graphs (DAGs)] [16, 17].

In the health sector, there are dedicated tools for assessing the risk of bias or internal validity [18, 19]. For example, Risk of Bias version 2 (RoB 2) [20] and Risk Of Bias In Non-randomised Studies—of Interventions (ROBINS-I) [21] are widely recommended domain-based tools for assessing the risk of bias in primary research (the former is for randomised controlled trials and the latter is for non-randomised intervention studies) when conducting systematic reviews in the health sector [18]. There is another widely recommended domain-based tool called Risk Of Bias In Systematic reviews (ROBIS) which is for specifically assessing the risk of bias arising from the conduct of secondary research in the health sector [18, 22]. These risk-of-bias assessment tools guide users to focus on a few ‘domains’ of bias (broad categories of bias specifically designed for assessing the risk of bias in particular situations; for example, when you plan to synthesise effect sizes from multiple studies, you would need to assess the risk of bias in primary research assessing effects by developing a comprehensive set of domains to address all potential sources of bias occurring in primary research in your subject area) [13]. Risk-of-bias assessors can make overall judgements about the extent of risk of bias for the specified domains (e.g., low risk of bias, some concerns, or high risk of bias [20]). The concept of domains of bias was initially developed in the health sector to assess the risk of bias for groups of types of bias that can occur at specific stages of primary or secondary research [20, 21]. For example, in the ROBINS-I tool, detection bias, recall bias, information bias, misclassification bias, observer bias, and measurement bias are all included in a domain called ‘risk of bias in measurement of outcome’ [21].

Following the developments of the risk-of-bias assessment tools in the health sector, the Collaboration for Environmental Evidence (hereafter ‘CEE’: environmentalevidence.org) is adopting such a domain-based approach and has been developing a domain-based critical appraisal tool called ‘CEE Critical Appraisal Tool’ since 2020. This tool is for assessing the risk of bias in primary research and is available for use in evidence reviews in the environmental sector (environmentalevidence.org/cee-critical-appraisal-tool). Employing domains of bias is now strongly encouraged when conducting evidence reviews in medical and health sciences [20, 21, 23, 24] as well as in the environmental sector [13] because assessing every individual type of bias is laborious and there are no universal definitions of all existing types of bias in estimation of causal effects in science. A recent review on critical appraisal in ecology highlighted this important development and the urgent need to evaluate the validity and reliability of the CEE Critical Appraisal Tool because, remarkably, only 4% of reviews labelled as ‘systematic reviews’ in ecology actually conducted critical appraisal [25]. This finding suggests insufficient education about biases in environmental science courses and a lack of understanding of how to avoid providing potentially biased estimates [26].

We aim to raise awareness about biases in the environmental sector by mapping types of bias that may impact the reliability of the results of environmental research evaluating the effects of interventions or exposures and categorising them into the developed domains of bias. The objectives of our research are: (1) to identify potential types of bias, that have been previously collated, in estimation of causal effects in either primary or secondary research; (2) to evaluate the relevance of the identified biases to the environmental sector, which incorporates not only non-human biotic (e.g., plants, animals) and abiotic subjects (e.g., water, atmosphere) but also human subjects; and (3) to utilise the resultant list of biases to evaluate the coverage of the domains of bias for a previously developed assessment tool to use in environmental research, namely the CEE Critical Appraisal Tool.

To avoid confusion, when we refer to domains of bias, we use plural rather than singular (e.g., confounding biases) as noted below. Also, note that we do not aim to evaluate relatedness among biases (e.g., we do not discuss whether selection bias is related to attrition bias [27]). Rather we aim to provide an overview of the types of bias from the view of managing the risk of bias by employing the developed domains.

## Methods

### Listing the types of bias previously collated and described elsewhere

In order to develop a comprehensive list of potential biases we used the Catalogue of Bias (catalogofbias.org), and reviewed 11 key publications in the field: Sackett [28], Bayliss and Beyer [29], Clarke et al. [30], Smith and Noble [31], Thakur et al. [32], Paradis [33], Warden [34], Delegado-Rodriguez and Llorca [35], Hartman et al. [36], Marchevsky [37], and Pannucci and Wilkins [38]. The Catalogue of Bias is an ongoing collaborative project aiming to map all the biases that affect health and medical evidence. The Catalogue of Bias platform was developed by the Centre for Evidence-Based Medicine (CEBM) at Oxford University through reviewing the literature and regular meetings. We considered the Catalogue of Bias to provide a robust and comprehensive source. To our knowledge, there was no equivalent database of bias in environmental sciences, so the Catalogue of Bias was the only choice, apart from the 11 key publications. The additional 11 key publications used as information sources were selected because they were known to the authors as seminal papers that collated and described biases. We listed biases that were described in these sources in a Microsoft Excel spreadsheet (Redmond, WA, USA). The intention here was to identify the breadth of biases that have been recognised or described and not to identify all studies describing each bias. We therefore did not conduct a systematic literature search. The broad scope of our review and the time and resources available also precluded this.

### Evaluating the relevance of the biases to estimation of effects in the environmental sector

**Fig. 1:**
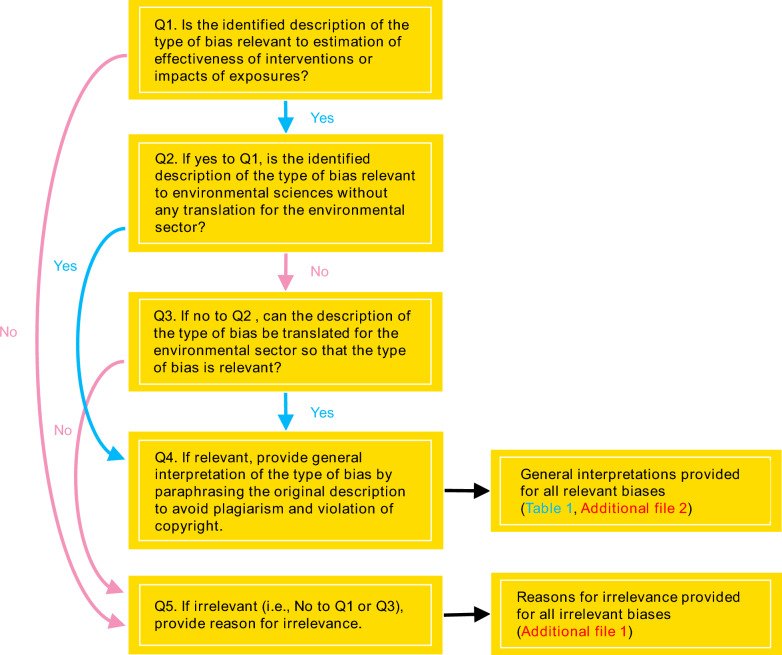
The algorithm used for evaluating the relevance of biases described in the sources

Since the vast majority of the types of bias were described in the medical and health research contexts, we evaluated them for relevance to estimation of effects of interventions or exposures in the environmental sector. Relevance was evaluated by a single reviewer (KK) using the algorithm provided in Fig. 1.

We employed a broad and all-inclusive scope for evaluating relevance to the environmental sector. The following a priori list of relevant topics was developed and used for assessing relevance: agriculture, aquaculture, biodiversity conservation, climate change, ecology and evolution, ecosystem services, environment and human wellbeing, environmental education, environmental legislation, fisheries, food security, forestry, invasive species, natural resource management, park and protected area management, pollution, soil management, sustainable energy use, waste management, wastewater management, water security, as well as any research relevant to environmental sustainability. The broad scope is consistent with that of CEE. The scope inherently included not only non-human biotic and abiotic subjects but also human subjects, however, human health outcomes were out of scope. The algorithm was applied by one person (KK) and the resulting decisions were checked by a second person (AP), and any disagreements or uncertainties were discussed among all authors. We highlight that we did not carry out any consistency checking, as we did not aim to aggregate effects, and did not aim to provide databases for aggregating effects. Hence, Cohen’s kappa statistic [39, 40] was not calculated.

### Evaluating the coverage of the developed domains of bias

**Fig. 2:**
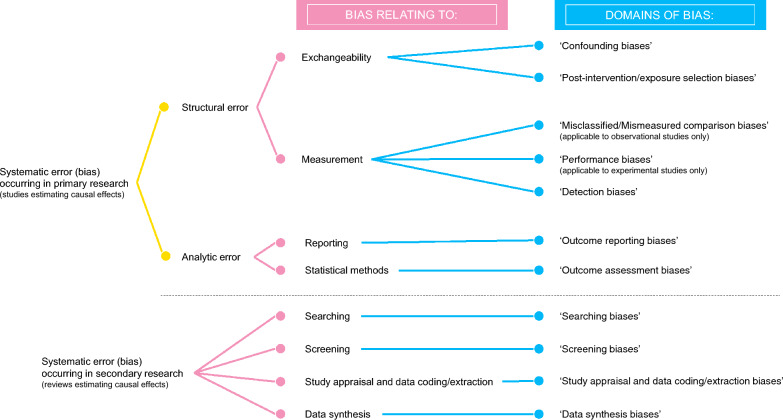
Organisation of bias from the broadest term (systematic error) to the developed domains of bias

Suzuki et al. [8] provided an organisational schema for systematic error in causal inference in primary research. In primary research, systematic error can be divided into structural error and analytic error. Structural error can be further divided into bias relating to measurement of intervention, exposure or outcome, and bias relating to exchangeability. Exchangeability refers to independence between the counterfactual outcome ${Y}^{a}$ (the outcome which a subject or area would have experienced if it had received a specified intervention or exposure value [41]) and the observed intervention or exposure $A$, and it is denoted as *Y*^*a*^ ⫫ A, for all treatment values *a* (all values of the treatment variable that may differ among individual subjects or areas) [42]. Alternatively, exchangeability refers to an assumption that the outcome in the comparator group would mirror the outcome in the intervention or exposure group if the subjects or areas in the comparator group had been subjected to the same intervention or exposure as those in the intervention or exposure group [17]. Analytic error is a systematic error that cannot be explained by structural error, and it includes bias relating to reporting [20, 21] and statistical methods [23, 24, 43]. Figure 2 illustrates our assumptions about the organisation of bias occurring in primary and secondary research from the broadest term (systematic error) to the developed domains of bias.

Frampton et al. [13] provided the principles of risk-of-bias assessment in the environmental sector and recommended seven domains (or classes) to be assessed when conducting evidence reviews assessing the effect of an intervention or exposure. These domains for primary research were consistent with the CEE Critical Appraisal Tool version 0.3 (see environmentalevidence.org/cee-critical-appraisal-tool for more details) as including the following:

1. Confounding biases: Risk of biases due to an uncontrolled (or inappropriately controlled) variable (confounder) that influences both the intervention/exposure and the outcome
2. Post-intervention/exposure selection biases: Risk of biases arising from systematic differences in the selection of subjects or areas into the study or analysis after the intervention or exposure
3. Misclassified/mismeasured comparison biases: Risk of biases arising from misclassification or mismeasurement of the intervention, exposure and/or comparator (applicable to observational studies only)
4. Performance biases: Risk of biases due to altered treatment procedure of interest (applicable to experimental studies only; treatment procedures are series of actions for applying the experimental (intervention or exposure) treatment [44]; note that an experimental treatment can be either an intervention or an exposure, or both, depending on the outcome measure. For example, when a fertilizer is applied to a crop, this is an intervention if the outcome measure is crop yield, but an exposure if the outcome measure is change in soil microfauna [45])
5. Detection biases: Risk of biases arising from systematic differences in measurement of outcomes
6. Outcome reporting biases: Risk of biases in reporting of study findings
7. Outcome assessment biases: Risk of biases due to error in applied statistical methods

Regarding the domains for secondary research, we used the CEE Synthesis Appraisal Tool (CEESAT) version 2 [46, 47]. CEESAT is an eight-criteria checklist consisting of sixteen questions for assessing environmental evidence reviews in terms of risk of bias, repeatability, and transparency, and hence not all the criteria are relevant to risk of bias (e.g., review question setting and provision of limitations). Using only the relevant criteria resulted in four domains of bias which were the same as the ROBIS tool [22], and thus consistent with a widely recommended tool in medical and health sciences [18]:

1. Searching biases: Risk of biases in searches of relevant records
2. Screening biases: Risk of biases arising from screening of potentially relevant records
3. Study appraisal and data coding/extraction biases: Risk of biases due to the lack of or inappropriate conduct of study appraisal and data coding/extraction
4. Data synthesis biases: Risk of biases due to employing inappropriate synthesis methods

Each type of bias was checked by a single reviewer (KK) for relevancy against each domain. Each bias-and-domain combination was data-coded in the database (spreadsheet), by the single reviewer using subjective judgements (‘Yes’ = relevant/‘No’ = not relevant). All decisions were double-checked by the other authors. The evaluator was allowed to take special notes when some types of bias were beyond the provided domains but there were no such cases. We also data-coded relevant levels of research (primary and/or secondary research) for all included types of bias.

## Results

### Types of bias relevant to the environmental sector

**Fig. 3:**
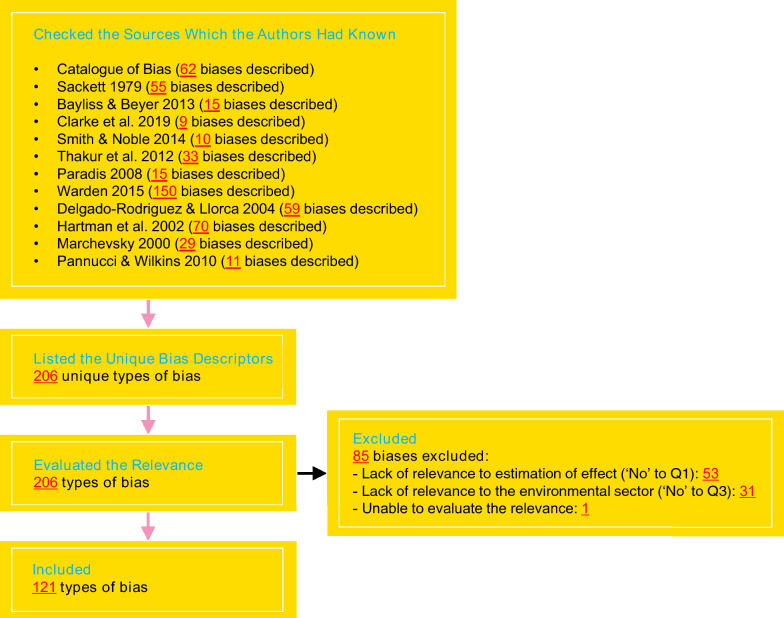
Identification of biases previously collated and described elsewhere

Figure 3 summarises the results of the bias selection process and provides the numbers of biases identified in each of the sources searched. Some biases were described by multiple sources while some biases were described by one source only. There were 206 types of bias after merging 53 bias descriptors that were specifically described as synonyms; we did not merge bias descriptors unless they were specifically referred to as synonyms to make sure none are missed from the list. Note that we did not reproduce (verbatim) the descriptions of the biases provided in the sources mainly due to copyright restrictions.

After the evaluation of relevance, we identified 121 types of bias (accompanied by 24 synonyms specified in the sources) that were relevant to estimation of effects in the environmental sector. Of the 85 excluded types of biases, 53 (62%) were excluded due to a lack of relevance to estimation of effects. Thirty-one (37%) were excluded due to a lack of relevance to the environmental sector; the most common reasons for exclusions were specifically referring to human health outcomes, diagnosis, and healthcare and medical interventions. One (1%; culture bias described by Warden 2015 [34]) was excluded because its description was insufficient for judging its relevance. All the excluded types of bias can be found in Additional file 1.

The general interpretations and relevant domains of the 121 types of bias relevant to the environmental sector are provided in Table 1. Sixty-eight types of bias (56%) were relevant to primary research only and 18 types of bias (15%) were relevant to secondary research only. Thirty-five types of bias (29%) were relevant to both the primary and secondary research. We provide the database of these biases in Additional file 2 so that readers can use the filter function of the spreadsheet to find types of bias relevant to specific domains. To increase its convenience, we also created a sheet to list types of bias relevant to each domain.

**Table 1:** Types of bias relevant to the environmental sector in the alphabetical order

	Types of bias	General interpretations	Bias occurring in*	Bias relating to**	Relevant domains of bias***	Source****
1	All’s well literature bias	Occurs when review papers deliberately omit or inadequately address studies that present contrasting results compared to studies yielding the results desired by certain individuals or groups of individuals	Secondary research	Screening Data synthesis	Screening biases Data synthesis biases	[28, 34, 36, 48]
2	Allocation bias (Allocation of intervention bias)	Occurs when non-random assignment of participants to study groups (i.e., allocation) creates an imbalance between the groups (i.e., exchangeability does not hold)	Primary research	Exchangeability	Confounding biases	[30, 35, 37, 49]
	Allocation of intervention bias	See 2 ‘Allocation bias’				
3	Allocation sequence bias	Occurs when the allocation sequence is not concealed to researchers and the selection creates an imbalance between the study groups (i.e., exchangeability does not hold)	Primary research	Exchangeability	Confounding biases	[34]
4	Analysis bias	Occurs when the knowledge of the results of a study causes inappropriate analyses and selective reporting	Primary research Secondary research	Reporting Statistical methods Data synthesis	Outcome reporting biases Outcome assessment biases Data synthesis biases	[30–32]
5	Ascertainment bias	Occurs when the ascertainment of subjects or areas included in a study or the ascertainment of measurements or collected data for analysis differs between the groups after the intervention/exposure	Primary research	Exchangeability Measurement	Post-intervention/exposure selection biases Misclassified/mismeasured comparison biases Performance biases Detection biases	[33, 35, 50]
6	Attention bias (Hawthorne effect)	Occurs when human or animal subjects change their behaviour due to the awareness of being observed by researchers	Primary research	Exchangeability Measurement	Confounding biases Performance biases	[28, 35, 37, 51]
7	Attrition bias	Occurs when missing data between groups are unequal, and exchangeability does not hold (cannot be assumed)	Primary research	Exchangeability	Post-intervention/exposure selection biases	[34, 52]
8	Authorization bias	Occurs when there is inability to obtain authorization for use of certain data or documents, and that causes deviation from a true effect	Primary research Secondary research	Exchangeability Searching	Confounding biases Post-intervention/exposure selection biases Searching biases	[36]
9	Availability bias	Occurs when the information that is readily available differs systematically from the information obtained by aggregating all pertinent data to answer the review question	Secondary research	Searching	Searching biases	[29, 53]
10	Bigwig bias	Occurs when information in documents written by high profile authors differs systematically from the wider evidence base relevant to the review question and is more likely to be cited as evidence in a review	Secondary research	Searching Screening Data synthesis	Searching biases Screening biases Data synthesis biases	[29]
11	Bogus control bias	Occurs when subjects or areas initially assigned to the intervention/exposure group are taken out from that group or moved to the control (no intervention/exposure) group and the assumption of exchangeability no longer holds	Primary research	Exchangeability	Confounding biases Post-intervention/exposure selection biases	[28, 34, 36]
12	Case definition bias	Occurs when the intervention/exposure is vaguely described or lacks relevant information, and the vagueness or the lack of information leads to mismeasurement of the intervention/exposure by researchers in an observational study	Primary research	Measurement	Misclassified/mismeasured comparison biases	[34]
13	Channelling bias	Occurs when researchers assign subjects or areas, that are more likely to respond to the intervention/exposure, to the intervention/exposure group, creating an imbalance between the groups (i.e., exchangeability does not hold)	Primary research	Exchangeability	Confounding biases	[33, 34, 38]
14	Chronological bias	Occurs when the timing of allocation of subjects or areas to a group (or groups) is different within or between groups and the time lag influences both the intervention/exposure and the outcome, or the subjects or areas are subject to a deviated intervention/exposure due to the time lag	Primary research	Exchangeability Measurement	Confounding biases Performance biases	[33, 38, 54]
15	Citation bias	Occurs when studies with statistically significant results are more likely to be cited as pieces of evidence in a review	Secondary research	Searching Screening Data synthesis	Searching biases Screening biases Data synthesis biases	[29, 33, 35, 38]
	Classification bias	See 52 ‘Information bias’				
16	Cognitive dissonance bias	Occurs when an investigator or a reviewer’s claim about an effect contradicts their obtained evidence due to the investigator or reviewer’s cognitive bias	Primary research Secondary research	Statistical methods Data synthesis	Outcome assessment biases Data synthesis biases	[28, 34]
17	Collider bias	Occurs when conditioning on a variable (i.e., filtering by a certain value of that variable) that is a common effect of the exposure and the outcome	Primary research	Exchangeability	Confounding biases Post-intervention/exposure selection biases	[55]
18	Compliance bias	Occurs when subjects compliant with the intervention differ from those not compliant in some characteristic and the characteristic is also associated with the outcome, or when non-compliance causes deviated initiation, implementation or discontinuation of the intervention between groups and the deviation is not taken into account	Primary research	Exchangeability Measurement	Confounding biases Post-intervention/exposure selection biases Performance biases	[32, 34–36, 56]
19	Confidentiality bias	Occurs when unrestricted research data, that systematically differs from restricted research data, are exclusively used due to commercial or privacy considerations	Primary research Secondary research	Exchangeability Searching	Confounding biases Searching biases	[29]
20	Confirmation bias	Occurs when the information to confirm ideas, beliefs or hypotheses is systematically different from the truth	Primary research Secondary research	Exchangeability Measurement Reporting Statistical methods Searching Screening Study appraisal and data coding/extraction Data synthesis	Confounding biases Post-intervention/exposure selection biases Misclassified/mismeasured comparison biases Performance biases Detection biases Outcome reporting biases Outcome assessment biases Searching biases Screening biases Study appraisal and data coding/extraction biases Data synthesis biases	[34, 57]
21	Conflict of interest	Occurs when a conflict of interest causes a systematic difference in estimation of effect	Primary research Secondary research	Exchangeability Measurement Reporting Statistical methods Searching Screening Study appraisal and data coding/extraction Data synthesis	Confounding biases Post-intervention/exposure selection biases Misclassified/mismeasured comparison biases Performance biases Detection biases Outcome reporting biases Outcome assessment biases Searching biases Screening biases Study appraisal and data coding/extraction biases Data synthesis biases	[33]
22	Confounding bias	Occurs when an uncontrolled (or inappropriately controlled) variable (confounder) that influences both the intervention/exposure and the outcome exists	Primary research	Exchangeability	Confounding biases	[31, 34, 37, 38, 58]
23	Confounding bias by group	Occurs when there is a variable describing communities (groups) and that influences both the intervention or exposure and the outcome	Primary research	Exchangeability	Confounding biases	[35]
24	Confounding bias by indication	Occurs when there exists an indication for the intervention/exposure (a preceding phenomenon that affects the intervention/exposure) or a variable influenced by this indication that affects both the intervention/exposure and the eventual outcome	Primary research	Exchangeability	Confounding biases	[35, 59]
25	Contamination bias	Occurs when subjects or areas in the comparator group (no or alternative intervention/exposure) accidentally receive the intervention/exposure (and vice versa), and this contamination leads to the violation of the assumption of exchangeability	Primary research	Exchangeability	Confounding biases	[28, 34–36]
26	Correlation bias	Occurs when equating non-causal association with causal association	Primary research Secondary research	Exchangeability Study appraisal and data coding/extraction Data synthesis	Confounding biases Study appraisal and data coding/extraction biases Data synthesis biases	[28, 32, 34, 36, 37]
	Cost bias	See 100 ‘Resource bias’				
27	Co-treatment bias	Occurs when inappropriate or unplanned co-treatment(s) is/are used in an experiment that results in confounding or altered treatment(s)	Primary research	Exchangeability Measurement	Confounding biases Performance biases	[30, 34]
28	Data capture bias (Data capture error)	Occurs when data capture (data collection and recording) errors favour or disfavour the outcome for the intervention/exposure group or the comparator group	Primary research Secondary research	Exchangeability Measurement Study appraisal and data coding/extraction	Confounding biases Post-intervention/exposure selection biases Misclassified/mismeasured comparison biases Performance biases Detection biases Study appraisal and data coding/extraction biases	[34]
	Data capture error	See 28 ‘Data capture bias’				
29	Data collection bias	Occurs when data collectors' beliefs influence the way relevant data are collected and that results in inaccurate estimation of an effect	Primary research Secondary research	Exchangeability Measurement Study appraisal and data coding/extraction	Confounding biases Post-intervention/exposure selection biases Misclassified/mismeasured comparison biases Performance biases Detection biases Study appraisal and data coding/extraction biases	[31]
	Data completeness bias	See 68 ‘Missing data bias’				
	Data entering bias	See 30 ‘Data entry bias’				
30	Data entry bias (Data entering bias)	Occurs when omitting data entry, inaccurately entering data, or inaccurately converting hand-written data into digital form	Primary research Secondary research	Exchangeability Measurement Statistical methods Study appraisal and data coding/extraction	Confounding biases Post-intervention/exposure selection biases Misclassified/mismeasured comparison biases Performance biases Detection biases Outcome assessment biases Study appraisal and data coding/extraction biases	[34, 37]
31	Data merging bias	Occurs when multiple data sets are merged incorrectly and the merged data produce an intentionally or unintentionally inaccurate estimate of an effect	Primary research Secondary research	Reporting Statistical methods Study appraisal and data coding/extraction	Outcome reporting biases Outcome assessment biases Study appraisal and data coding/extraction biases	[34]
32	Data-dredging bias (‘Looking for the pony’)	Occurs when the results of inappropriate statistical analyses or synthesis methods, that are not pre-specified, are presented, or statistical or synthesis methods are used inappropriately to support claims	Primary research Secondary research	Reporting Statistical methods Data synthesis	Outcome reporting biases Outcome assessment biases Data synthesis biases	[28, 32, 34, 36, 60]
33	Definition bias	Occurs when definitions of important words used in a study or a review are not accurate or precise so that misinterpretation affects estimation of an effect	Primary research Secondary research	Exchangeability Measurement Searching Screening Study appraisal and data coding/extraction	Confounding biases Post-intervention/exposure selection biases Misclassified/mismeasured comparison biases Performance biases Detection biases Searching biases Screening biases Study appraisal and data coding/extraction biases	[32]
34	Design bias	Occurs when inferior experiments or treatments, reflecting a desire for certain results, are employed	Primary research	Exchangeability Measurement	Confounding biases Post-intervention/exposure selection biases Performance biases Detection biases	[30, 31, 33, 34]
35	Detection bias	Occurs when there are systematic differences in measurement of outcomes between groups	Primary research	Measurement	Detection biases	[15, 33–36]
36	Differential maturing bias	Occurs when there are uneven secular trends (long-term and sustained changes or patterns) between the groups due to differential maturing, and that creates an imbalance between the groups before the intervention or exposure (time-varying confounding)	Primary research	Exchangeability	Confounding biases	[35]
37	Dissemination bias	Occurs when retrieval of information does not result in a representative sample of studies due to the way study results are disseminated, or when study results affect reporting or dissemination	Primary research Secondary research	Reporting Searching	Outcome reporting biases Searching biases	[35]
38	Distribution assumption bias	Occurs when a data analyst conducts a statistical test under a false assumption of the data distribution or the approach is not justifiable so that the inference is invalid	Primary research	Statistical methods	Outcome assessment biases	[34]
	Dropout bias	See 120 ‘Withdrawal bias’				
39	Duplication bias (Multiple publication bias)	Occurs when duplicated research findings are included and synthesised in a review	Secondary research	Screening	Screening biases	[29, 37]
40	End-digit preference bias	Occurs when there are systematic differences in quantitative measurements due to a preference for a certain ending digit (e.g., 0 by rounding up/down rather than retaining the original digits), typically ending with an unusual frequency	Primary research Secondary research	Statistical methods Study appraisal and data coding/extraction	Outcome assessment biases Study appraisal and data coding/extraction biases	[28, 32, 34, 36]
41	Estimator bias	Occurs when an inappropriate estimator (a statistic used to estimate the parameter) is used for estimating the true causal effect (parameter)	Primary research Secondary research	Statistical methods Data synthesis	Outcome assessment biases Data synthesis biases	[34]
42	Exclusion bias	Occurs when the criteria for inclusion of subjects or areas into a study or analysis are applied differently to the intervention/exposure group and the comparator group	Primary research	Exchangeability	Confounding biases Post-intervention/exposure selection biases	[34, 35, 37]
43	Expectation bias	Occurs when observers err in measurement, recording, or data collection due to prior expectations	Primary research Secondary research	Exchangeability Measurement Searching Screening Study appraisal and data coding/extraction	Confounding biases Post-intervention/exposure selection biases Misclassified/mismeasured comparison biases Performance biases Detection biases Searching biases Screening biases Study appraisal and data coding/extraction biases	[28, 34, 36, 37]
44	Exposure suspicion bias	Occurs when a knowledge or awareness of the subject's or area's exposure status influences selection, measurement, reporting, or analysis	Primary research	Exchangeability Measurement Reporting Statistical methods	Confounding biases Post-intervention/exposure selection biases Misclassified/mismeasured comparison biases Performance biases Detection biases Outcome reporting biases Outcome assessment biases	[28, 35, 36]
45	Familiarity bias	Occurs when reviewers limit searching to documents relevant to their own discipline	Secondary research	Searching	Searching biases	[29]
46	Foreign language exclusion bias	Occurs when language is restricted in a review and studies with certain direction of results or certain topics are more or less likely to be disseminated in the eligible language(s)	Secondary research	Searching Screening	Searching biases Screening biases	[34]
47	Friend control bias	Occurs when there is an association of the actual intervention/exposure status between human subjects in the intervention/exposure group and their friends in the comparator group due to mismeasurements, misclassifications of or the lack of adherence to the intervention/exposure	Primary research	Measurement	Performance biases	[35]
	Handling data bias	See 68 ‘Missing data bias’				
	Hawthorne effect	See 6 ‘Attention bias’				
48	Hot stuff bias	Occurs when research results on hot topics are published even though they are not valid	Primary research Secondary research	Exchangeability Measurement Reporting Statistical methods Searching Screening Study appraisal and data coding/extraction Data synthesis	Confounding biases Post-intervention/exposure selection biases Misclassified/mismeasured comparison biases Performance biases Detection biases Outcome reporting biases Outcome assessment biases Searching biases Screening biases Study appraisal and data coding/extraction biases Data synthesis biases	[28, 34, 36, 61]
49	Inclusion bias	Occurs when inclusion of subjects or areas into a study creates an imbalance between the groups (i.e., exchangeability does not hold)	Primary research	Exchangeability	Confounding biases Post-intervention/exposure selection biases	[31, 35]
50	Inclusion control bias	Occurs when some subjects or areas of the comparator group also receive the intervention/exposure to some extent (biased towards null when there is an effect)	Primary research	Exchangeability	Confounding biases Post-intervention/exposure selection biases	[34]
51	Industry sponsorship bias	Occurs when research outcomes are intentionally aligned with the interests of the funding commercial manufacturer, undermining the validity of the inference	Primary research Secondary research	Exchangeability Measurement Reporting Statistical methods Searching Screening Study appraisal and data coding/extraction Data synthesis	Confounding biases Post-intervention/exposure selection biases Misclassified/mismeasured comparison biases Performance biases Detection biases Outcome reporting biases Outcome assessment biases Searching biases Screening biases Study appraisal and data coding/extraction biases Data synthesis biases	[36, 62]
52	Information bias (Observation bias, Classification bias)	Occurs when the information used in a study deviates from the truth	Primary research	Exchangeability Measurement	Confounding biases Post-intervention/exposure selection biases Misclassified/mismeasured comparison biases Performance biases Detection biases	[32–34, 63]
53	Insensitive measure bias	Occurs when changes or differences in the outcome of interest are not detected accurately due to insufficient methods or inappropriate choice of an outcome variable	Primary research	Measurement	Detection biases	[28, 32, 34, 36, 64]
54	Instrument bias	Occurs when instruments used for measurement are inaccurate, too imprecise or different between groups (hence not comparable between groups)	Primary research	Exchangeability Measurement	Confounding biases Misclassified/mismeasured comparison biases Performance biases Detection biases	[28, 32, 34, 36]
55	Interobserver variability bias	Occurs when multiple observers' measurements of the intervention/exposure or the outcome are different, and the differences influence the estimate of an effect	Primary research	Measurement	Misclassified/mismeasured comparison biases Performance biases Detection biases	[34]
56	Interpretation bias	Occurs when researchers misinterpret the obtained data and results	Primary research Secondary research	Statistical methods Study appraisal and data coding/extraction Data synthesis	Outcome assessment biases Study appraisal and data coding/extraction biases Data synthesis biases	[32, 34]
57	Intra-observer variability bias	Occurs when an observer measures the intervention/exposure or the outcome differently and the differences influence the estimate of an effect	Primary research	Measurement	Misclassified/mismeasured comparison biases Performance biases Detection biases	[34]
58	Investigator bias	Occurs when study investigators (measurers) are not blinded and the lack of blinding influences estimation of an effect	Primary research	Exchangeability Measurement	Confounding biases Post-intervention/exposure selection biases Misclassified/mismeasured comparison biases Performance biases Detection biases	[37]
59	Laboratory data bias	Occurs when procedures implemented at a laboratory produce a systematic error in the estimate of an effect (may be confirmed by comparing data from multiple laboratories)	Primary research	Exchangeability Measurement	Confounding biases Post-intervention/exposure selection biases Performance biases Detection biases	[34]
60	Lack of blinding bias	Occurs when the lack of concealment of the intervention, exposure or control received by subjects or areas affects selection of subjects, areas or data, measurement of the treatment or the outcome, and/or assessment of the effectiveness or the impact	Primary research	Exchangeability Measurement Statistical methods	Post-intervention/exposure selection biases Misclassified/mismeasured comparison biases Performance biases Detection biases Outcome assessment biases	[65]
	Lack of intention to treat analysis bias	See 117 ‘Treatment analysis bias’				
61	Language bias	Occurs when studies with certain results or topics are more likely to be published in a certain language	Secondary research	Searching Screening	Searching biases Screening biases	[29, 35, 66]
62	Latency bias	Occurs when the outcome is assessed in an inappropriate time interval (i.e., the time interval is too short or long) for the intended assessment of causal effect	Primary research Secondary research	Statistical methods Data synthesis	Outcome assessment biases Data synthesis biases	[34]
63	Literature search bias	Occurs when searching of literature does not capture a representative sample of all existing studies relative to the review question	Secondary research	Searching	Searching biases	[34]
64	Location bias	Occurs when some journals are not indexed in all databases so that the choice of databases influences results of literature searching	Secondary research	Searching	Searching biases	[29]
	‘Looking for the pony’	See 32 ‘Data-dredging bias’				
	Loss to follow-up bias	See 120 ‘Withdrawal bias’				
65	Measurement bias	Occurs when measurements of relevant variables are not accurate or precise enough	Primary research	Measurement	Misclassified/mismeasured comparison biases Performance biases Detection biases	[31]
66	Meta-analysis bias	Occurs when meta-analyses based on a non-representative sample of studies are conducted	Primary research Secondary research	Searching Screening Study appraisal and data coding/extraction Data synthesis	Searching biases Screening biases Study appraisal and data coding/extraction biases Data synthesis biases	[36]
67	Misclassification bias	Occurs when subjects or areas, or the exposure or intervention is misclassified, and that distorts the association between the intervention/exposure and the outcome	Primary research	Exchangeability Measurement	Confounding biases Post-intervention/exposure selection biases Misclassified/mismeasured comparison biases Performance biases	[32, 34–36, 38, 67]
68	Missing data bias (Handling data bias, Data completeness bias)	Occurs when there is an imbalance in missing data between the comparison groups	Primary research	Exchangeability	Post-intervention/exposure selection biases	[34]
69	Misuse of statistics	Occurs when descriptive or inferential statistical analysis is inappropriately conducted, resulting in unjustified and invalid conclusions about the estimate of an effect	Primary research Secondary research	Statistical methods Data synthesis	Outcome assessment biases Data synthesis biases	[37]
70	Multiple exposure bias	Occurs when another exposure acts as a confounder affecting both the exposure of interest and the outcome	Primary research	Exchangeability	Confounding biases	[34]
	Multiple publication bias	See 39 ‘Duplication bias’				
	Neyman bias	See 89 ‘Prevalence-incidence bias’				
71	Non-contemporaneous control bias (Non-simultaneous comparison bias)	Occurs when there is a systematic difference in the timing of observation or investigation between the intervention/exposure and the control (or alternative intervention/exposure), and exchangeability between groups cannot be assumed due to the time difference	Primary research	Exchangeability	Confounding biases	[28, 34, 36, 37, 68]
72	Non-random sampling bias	Occurs when non-random sampling results in an imbalance between the intervention/exposure and the comparator groups (i.e., exchangeability does not hold)	Primary research	Exchangeability	Confounding biases Post-intervention/exposure selection biases	[34, 35, 37]
73	Non-respondent bias	Occurs when there is unequal loss of human subjects between the groups due to non-responses, or when there is a systematic difference in outcome measurement between the groups due to late and/or early responses	Primary research	Exchangeability Measurement	Post-intervention/exposure selection biases Detection biases	[28, 35, 37, 69]
74	Non-specification bias	Occurs when the intended intervention or exposure is not clearly defined, and an unintentional intervention or exposure occurs that influences both the intended intervention or exposure and the outcome	Primary research	Exchangeability	Confounding biases	[36]
	Non-simultaneous comparison bias	See 71 ‘Non-contemporaneous control bias’				
75	Novelty bias	Occurs when the interventions or exposures are reported to have a greater effect simply because they are novel	Primary research Secondary research	Exchangeability Measurement Reporting Statistical methods Searching Screening Study appraisal and data coding/extraction Data synthesis	Confounding biases Post-intervention/exposure selection biases Misclassified/mismeasured comparison biases Performance biases Detection biases Outcome reporting biases Outcome assessment biases Searching biases Screening biases Study appraisal and data coding/extraction biases Data synthesis biases	[70]
76	Obsequiousness bias	Occurs when human subjects alter questionnaire answers due to perception of the desired direction of the outcome held by investigators	Primary research	Measurement	Detection biases	[28, 34–37]
	Observation bias	See 52 ‘Information bias’				
77	Observer bias	Occurs when there are systematic differences in the process of observing and recording information between the groups in primary research	Primary research	Exchangeability Measurement	Confounding biases Post-intervention/exposure selection biases Misclassified/mismeasured comparison biases Performance biases Detection biases	[14, 34, 35]
78	Omitted-variable bias	Occurs when one or more important explanatory variables are not included in a regression model where omission of such variables is not appropriate for estimating an effect	Primary research	Statistical methods	Outcome assessment biases	[34, 35]
79	One-sided reference bias	Occurs when citing references to only those supporting one side of available evidence and hence systematically deviating from the truth	Secondary research	Searching Screening	Searching biases Screening biases	[28, 34, 36, 71]
80	Optimism bias	Occurs when researchers' or study subjects' beliefs that new interventions are better than established ones influence a study or review	Primary research Secondary research	Exchangeability Measurement Reporting Statistical methods Searching Screening Study appraisal and data coding/extraction Data synthesis	Confounding biases Post-intervention/exposure selection biases Misclassified/mismeasured comparison biases Performance biases Detection biases Outcome reporting biases Outcome assessment biases Searching biases Screening biases Study appraisal and data coding/extraction biases Data synthesis biases	[33]
81	Outcome reporting bias	Occurs when outcomes are selectively reported in a published document	Primary research	Reporting	Outcome reporting biases	[72]
	Outlier handling bias	See 115 ‘Tidying-up bias’				
82	Overmatching bias	Occurs when researchers match by (also ‘block on’, meaning filter by a certain value of that variable) a non-confounding variable that is associated with the intervention/exposure but not to the outcome	Primary research	Exchangeability	Confounding biases	[34, 35]
83	Perception bias	Occurs when relevant information or subjects, areas, interventions, exposures, controls, alternative interventions, alternative exposures or outcomes are misinterpreted in a study or review	Primary research Secondary research	Exchangeability Measurement Reporting Statistical methods Searching Screening Study appraisal and data coding/extraction Data synthesis	Confounding biases Post-intervention/exposure selection biases Misclassified/mismeasured comparison biases Performance biases Detection biases Outcome reporting biases Outcome assessment biases Searching biases Screening biases Study appraisal and data coding/extraction biases Data synthesis biases	[73]
84	Performance bias (Procedure bias)	Occurs when the treatment procedure of interest is altered without changing the inferential goal in an experimental study	Primary research	Measurement	Performance biases	[32–34, 38, 74]
85	Positive results bias	Occurs when positive results are more likely to be disseminated or submitted and accepted for publication than non-significant or negative results	Primary research Secondary research	Reporting Searching Screening	Outcome reporting biases Searching biases Screening biases	[28, 34, 36, 75]
	Post hoc analysis bias	See 86 ‘Post hoc analysis bias’				
86	Post-hoc significant bias (Post hoc analysis bias)	Occurs when researchers choose a critical significance level (α, 0.05 is commonly employed), and/or set a non-directional (two-tailed) or directional (one-tailed) hypothesis after some examination of data in an attempt to yield significant results	Primary research Secondary research	Statistical methods Data synthesis	Outcome assessment biases Data synthesis biases	[28, 32, 34–37]
87	Pre-publication bias	Occurs when previously published research, that is errant, is used to support a particular outcome in primary research	Primary research	Exchangeability Measurement Reporting Statistical methods	Confounding biases Post-intervention/exposure selection biases Misclassified/mismeasured comparison biases Performance biases Detection biases Outcome reporting biases Outcome assessment biases	[30]
88	Prevailing paradigm bias	Occurs when studies that are relevant to or support prevailing paradigms are more likely to be published in academic journals	Primary research Secondary research	Reporting Searching Screening	Outcome reporting biases Searching biases Screening biases	[29]
89	Prevalence-incidence bias (Neyman bias, Selective survival bias)	Occurs when uneven exclusion or attrition of subjects or areas with severe or mild responses occurs between the groups, and the assumption of exchangeability no longer holds	Primary research	Exchangeability	Post-intervention/exposure selection biases	[28, 32, 34–37, 76]
	Procedure bias	See 84 ‘Performance bias’				
90	Proficiency bias	Occurs when the intervention/exposure under study is unequally applied to individual subjects or areas due to interpersonal or intrapersonal differences	Primary research	Measurement	Performance biases	[32, 34, 36]
91	Publication bias	Occurs when the likelihood of a study being accepted for publication is influenced by the results of the study	Primary research Secondary research	Reporting Searching Screening	Outcome reporting biases Searching biases Screening biases	[29, 31, 32, 35, 37, 77]
92	Recall bias	Occurs when recall of relevant events or experiences by human subjects is inaccurate or incomplete	Primary research	Measurement	Misclassified/mismeasured comparison biases Detection biases	[32–38, 78]
93	Reference bias	Occurs when studies referenced as evidence in a review are not a representative sample of all existing studies relative to the review question	Secondary research	Searching Screening Data synthesis	Searching biases Screening biases Data synthesis biases	[29]
94	Regression dilution bias	Occurs when imprecise measurements of the intervention/exposure are used to estimate the effect of the intervention/exposure in an observational study	Primary research	Measurement	Misclassified/mismeasured comparison biases	[35]
95	Relative control bias	Occurs when there is an association of the actual intervention/exposure status between the intervention/exposure group and the comparator group due to mismeasurements, misclassifications of or the lack of adherence to the intervention/exposure	Primary research	Measurement	Misclassified/mismeasured comparison biases Performance biases	[35]
96	Repeated peeks bias	Occurs when repeated peeks at accumulating data in a study or review leads to inappropriate termination of data collection	Primary research Secondary research	Exchangeability Measurement Searching Screening Study appraisal and data coding/extraction	Confounding biases Post-intervention/exposure selection biases Misclassified/mismeasured comparison biases Performance biases Detection biases Searching biases Screening biases Study appraisal and data coding/extraction biases	[28, 34, 36]
97	Reporting bias	Occurs when certain results of a study or certain studies are more likely to be reported or unreported	Primary research Secondary research	Reporting Searching Screening	Outcome reporting biases Searching biases Screening biases	[30, 34, 79]
98	Research bias	Occurs when studies are carried out and reported on specific organisms or systems, or in particular conditions, because of varying levels of practicality or the anticipation of specific outcomes	Primary research Secondary research	Reporting Searching Screening	Outcome reporting biases Searching biases Screening biases	[29]
99	Researcher bias (Sponsor bias)	Occurs when fraud or manipulation of research design, data or results occurs due to vested interests of researchers and organisations	Primary research Secondary research	Exchangeability Measurement Reporting Statistical methods Searching Screening Study appraisal and data coding/extraction Data synthesis	Confounding biases Post-intervention/exposure selection biases Misclassified/mismeasured comparison biases Performance biases Detection biases Outcome reporting biases Outcome assessment biases Searching biases Screening biases Study appraisal and data coding/extraction biases Data synthesis biases	[30]
100	Resource bias (Cost bias)	Occurs when available budget or resources limit searching for evidence, resulting in a systematic difference between the identified and missed studies	Secondary research	Searching	Searching biases	[29]
101	Response fatigue bias	Occurs when study subjects suffer from fatigue due to implementation of the intervention/exposure and their responses are not obtained or they did not go through the entire process of the intended intervention/exposure	Primary research	Exchangeability Measurement	Post-intervention/exposure selection biases Performance biases	[34]
102	Review bias	Occurs when a researcher's prior knowledge or belief triggers misinterpretation of available data on the intervention/exposure or the outcome in primary research	Primary research	Measurement	Misclassified/mismeasured comparison biases Performance biases Detection biases	[34]
103	Reviewer bias	Occurs when the selection of studies from the available evidence is not a representative sample of all existing studies relative to the review question	Secondary research	Searching Screening Study appraisal and data coding/extraction Data synthesis	Searching biases Screening biases Study appraisal and data coding/extraction biases Data synthesis biases	[30]
	Rumination bias	See 118 ‘Underlying cause bias’				
	Sample size bias	See 121 ‘Wrong sample size bias’				
104	Sampling bias	Occurs when exchangeability between groups does not hold due to employed sampling techniques	Primary research	Exchangeability	Confounding biases Post-intervention/exposure selection biases	[34]
105	Scale degradation bias	Occurs when researchers make outcome measurement scales less precise or accurate to obscure differences between groups under comparison	Primary research Secondary research	Measurement Study appraisal and data coding/extraction	Detection biases Study appraisal and data coding/extraction biases	[28, 34, 36]
106	Selection bias	Occurs when exchangeability between groups does not hold due to the way that selection of subjects or areas into the study or analysis is carried out	Primary research	Exchangeability	Confounding biases Post-intervention/exposure selection biases	[33–35, 37, 38, 80]
	Selective survival bias	See 89 ‘Prevalence-incidence bias’				
107	Sick quitter bias	Occurs when the exposure or intervention that specific subjects receive is inaccurately measured or classified due to changes in the subjects' behaviour caused by sickness (e.g., lack of adherence, reclassification from intervention/exposure to no intervention/exposure by the researchers after the intervention/exposure is applied)	Primary research	Measurement	Misclassified/mismeasured comparison biases Performance biases	[35]
108	Significance bias	Occurs when statistical significance is confused with environmental, ecological, biological or agricultural significance	Secondary research	Study appraisal and data coding/extraction Data synthesis	Study appraisal and data coding/extraction biases Data synthesis biases	[28, 32, 34, 36, 37]
109	Spatial bias	Occurs when populations that are spatially distinct are compared, and this spatial difference affects both the intervention/exposure and the outcome being studied	Primary research	Exchangeability	Confounding biases	[34]
110	Spin bias	Occurs when research findings or data are inappropriately used or interpreted to support or oppose effectiveness or impact	Primary research Secondary research	Reporting Statistical methods Screening Study appraisal and data coding/extraction Data synthesis	Outcome reporting biases Outcome assessment biases Screening biases Study appraisal and data coding/extraction biases Data synthesis biases	[81]
	Sponsor bias	See 99 ‘Researcher bias’				
111	Starting time bias	Occurs when starting time for the intervention/exposure or outcome measurement is different within or between groups and the time lag (or variable influenced by the time lag) influences both the intervention/exposure and the outcome or causes inaccurate measurements of the intervention/exposure or the outcome	Primary research	Exchangeability Measurement	Confounding biases Misclassified/mismeasured comparison biases Performance biases Detection biases	[28, 34, 36, 82]
112	Substitution game bias	Occurs when substitution of the outcome or the intervention/exposure with a surrogate is imprecise or inaccurate	Primary research	Measurement	Misclassified/mismeasured comparison biases Performance biases Detection biases	[28, 34, 36, 83]
113	Susceptibility bias (Vulnerability bias)	Occurs when different study groups have different characteristics and the difference indicates that one group is more or less susceptible to the exposure (i.e., the characteristics are associated with the exposure and thus the difference creates an imbalance between the groups, meaning exchangeability does not hold)	Primary research	Exchangeability	Confounding biases Post-intervention/exposure selection biases	[33–36]
114	Temporal bias	Occurs when studies with smaller *p*-values or larger effects are more likely to be published in academic journals more rapidly	Secondary research	Searching Screening	Searching biases Screening biases	[29]
115	Tidying-up bias (Outlier handling bias)	Occurs when outliers or other untidy measurements or results are inappropriately excluded or non-reported	Primary research Secondary research	Reporting Statistical methods Study appraisal and data coding/extraction Data synthesis	Outcome reporting biases Outcome assessment biases Study appraisal and data coding/extraction biases Data synthesis biases	[28, 32, 34, 36, 37]
116	Transfer bias	Occurs when a certain aspect of a study leads to uneven losses in follow-up between the groups	Primary research	Exchangeability	Post-intervention/exposure selection biases	[32, 33, 38]
117	Treatment analysis bias (Lack of intention to treat analysis bias)	Occurs when researchers fail to keep subjects or areas in the group they are assigned to, when there is deviation from the intended treatment, or when subjects do not adhere to the assigned treatment	Primary research	Exchangeability Measurement	Confounding biases Performance biases	[34, 35]
118	Underlying cause bias (Rumination bias)	Occurs when the intervention or exposure (group) is more carefully planned and implemented than the control (no intervention or exposure), and that results in imbalances between the groups or misclassification or mismeasurement of control	Primary research	Exchangeability Measurement	Confounding biases Misclassified/mismeasured comparison biases	[28, 32, 34–36]
119	Volunteer bias	Occurs when human subjects involved in a study differ in some way among those in the intervention/exposure group or between groups due to willingness to participate as volunteers	Primary research	Exchangeability Measurement	Confounding biases Post-intervention/exposure selection biases Performance biases	[28, 32, 34, 36, 37, 84]
	Vulnerability bias	See 113 ‘Susceptibility bias’				
120	Withdrawal bias (Dropout bias, Loss to follow-up bias)	Occurs when subjects who have dropped out of a study differ from remaining subjects, and the differences modify the effectiveness or impact	Primary research	Exchangeability	Confounding biases Post-intervention/exposure selection biases	[28, 32, 34–37]
121	Wrong sample size bias (Sample size bias)	Occurs when used sample size is inappropriate in a study or analysis for assessing the effect of the intervention or exposure due to violation of the assumption that exchangeability holds or the hypothesis can be tested with the collected samples	Primary research	Exchangeability Statistical methods	Confounding biases Post-intervention/exposure selection biases Outcome assessment biases	[28, 34, 36, 85]

Synonyms are provided in brackets. Note that these general interpretations assume that no statistical corrections or adjustments are made for providing valid estimates

^*^Primary research and/or secondary research. **Selected from the following items which are also provided in Fig. 2: (1) exchangeability; (2) measurement; (3) reporting; (4) statistical methods; (5) searching; (6) screening; (7) study appraisal and data coding/extraction; and (8) data synthesis. ***Domains are (1) confounding biases; (2) post-intervention/exposure selection biases; (3) misclassified/mismeasured comparison biases; (4) performance biases; (5) detection biases; (6) outcome reporting biases; (7) outcome assessment biases; (8) searching biases; (9) screening biases; (10) study appraisal and data coding/extraction biases; and (11) data synthesis biases. ****Documents and specific webpages of Catalogue of Bias that provided descriptions of the biases

### The coverage of the domains of bias

There was no type of bias that was not applicable to any domain, and we did not find any types of bias that were beyond the domains, suggesting that domains were sufficient to cover the 121 types of bias. Eighty-six types of bias (71%) were applicable to multiple domains while 35 types of bias (29%) were applicable to only one domain of bias.

## Discussion

### Overview of findings

We found 121 (out of 206) types of bias that were relevant to estimation of causal effects in the environmental sector. We provided a general interpretation of every relevant type of bias covered by seven risk-of-bias domains for primary research and four domains for secondary research.

### Strengths and limitations

As far as we are aware this is the first study to provide a comprehensive map of the type of bias that may affect the reliability of the results of environmental research studies and reviews. It provides an important summary of the breadth of biases which may impact the findings. There are limitations that should be noted. We used a pragmatic approach to identify potentially relevant biases by utilising the Catalogue of Bias and 11 key publications. We did not conduct a systematic literature search, similar to the approach used by some of the key documents we consulted [28–33, 35–38]. However, the Catalogue of Bias which is developed by CEBM at Oxford University and is continually updated, is considered a robust source for identifying biases that affect health and medical evidence. There may be other relevant types of bias that are collated and described elsewhere, but we are not aware of any published studies specific to the environmental sector that aimed to identify types of bias. We used subjective judgements for evaluating relevance and some aspects of biases were open for interpretation although we conducted independent double checking. We provided only ‘general’ interpretations of the types of bias in this paper.

### Implications for research and practice

Since education about biases is not sufficient and the level of knowledge about biases are concerning in the environmental sector [26], we hope the provision of the general interpretations of the 121 types of bias helps environmental scientists, as well as decision-makers, be better aware of the biases. When environmental scientists, practitioners, and policymakers come across certain types of bias when they read research or review papers, or when they communicate about certain types of bias, we suggest they check our dedicated list of biases relevant to estimation of causal effects in the environmental sector.

Although some types of bias such as confounding bias, selection bias, and measurement bias have been formally defined [8, 17], future research is needed to formalise definitions of other types of bias such as through decomposition using mathematical formulae and/or by employing directed acyclic graphs (DAGs). For example, Suzuki et al. 2016 [8] decomposed confounding bias by comparing the causal measure (A → Y), *µ*(*E*(*Y*_1_), *E*(*Y*_0_)), and the situation where a confounding variable is present as:

$$\mu \left( {E\left( {Y{|}A = 1} \right), E\left( {Y{|}A = 0} \right)} \right) - \mu \left( {E\left( {Y_{1} } \right),E\left( {Y_{0} } \right)} \right)$$

where *Y* refers to outcome and *A* refers to intervention or exposure (*A* = 1 receives the intervention or exposure and *A* = 0 does not receive the intervention or exposure). Although the practicality of defining all biases remains to be evaluated, formalising definitions is particularly important because, to some extent, vagueness remains in the descriptions of the biases. This would also help identify duplication and cross over between biases identified by different researchers.

As the types of bias are described theoretically, future research is also needed for collating empirical studies that evaluate the impacts of the identified biases. Such research is desired even in the health sector for better managing the risk of bias [86]. In the environmental sector, for example, Hudson et al. [87] and Takeshita et al. [88] evaluated the potential extent of selection bias and confounding bias, respectively. Konno et al. [89] and Konno and Pullin [90] evaluated the potential extent of language bias and availability bias, respectively. If such studies are collated and summarised, environmental scientists should be better able to manage the risk of bias. We hope this paper guides future research.

A lack of and inappropriate conduct of risk-of-bias assessment in the environmental sector has been reported in the literature [13, 25, 91]. Our findings showed that the domains selected for use in CEE Critical Appraisal Tool and CEE Synthesis Assessment Tool are sufficient and cover all the biases identified in primary and secondary research (Fig. 2) and can be used to facilitate communication about the risk of bias. A domain-based tool should incorporate a series of checklist questions within each domain that prompts users to consider relevant types and sources of bias. Developing a series of checklist questions requires relevant expertise [20, 21], and thus, we encourage evidence providers (e.g., impact evaluators, evidence synthesists) as well as evidence consumers (e.g., decision-makers) to make use of the existing tools for assessing the risk of bias (e.g., the CEE Critical Appraisal Tool for primary research and the CEE Synthesis Appraisal Tool for secondary research), rather than developing a tool on their own, as it may result in inappropriate conduct of risk-of-bias assessment [13, 91]. However, at the time of writing, the CEE Critical Appraisal Tool is still under development, and we hope users of this tool will provide their feedback for the developers. The most important aspect for further development of the tool is inter-rater reliability (i.e., consistency between assessors) which can be quantified using a kappa statistic [92] and reported in eventual publications (e.g., Systematic Reviews [93], Systematic Maps [94]). Even in medical and health sciences, inter-rater reliability is still an issue [95, 96].

As part of our future work, we plan to consult researchers and policymakers to reach a consensus on the list of biases for better and active communication within the communities. We highlight the importance of including appropriately qualified and experienced stakeholders in any consultation because it is likely that many people working in environmental research and policy are inadequately familiar with biases [26], and thus there is a need to educate environmental scientists and decision-makers about the types of bias. There is also a need to provide training and develop capacity for use of risk-of-bias assessment tools in the environmental sciences. We are not aware of any institution that teaches this subject in environmental contexts, even at Masters level. In response to this gap, CEE intends to develop a series of online training videos on critical appraisal. If many more scholars, institutions as well as learned societies can help the environmental sector better understand the nature of biases, more active communication of the risk of bias may be achieved.

## Supplementary Information

**Additional file 1.** Excluded types of bias.

**Additional file 2.** Included types of bias.

## Data Availability

The datasets generated during the current study are available in Additional files.
